# Plant water use characteristics of five dominant shrub species of the Lower Rio Grande Valley, Texas, USA: implications for shrubland restoration and conservation

**DOI:** 10.1093/conphys/cou005

**Published:** 2014-02-18

**Authors:** Arjun Adhikari, Joseph D. White

**Affiliations:** 1 The Department of Biology, Baylor University, One Bear Place #97388, Waco, TX 76798, USA; 2 The Institute of Ecological, Earth, and Environmental Sciences, Baylor University, One Bear Place #97205, Waco, TX 76798, USA

**Keywords:** Gas exchange, soil salinity, soil water deficit, soil water potential, thorn shrubs

## Abstract

The biogeographic distribution of plant species is inherently associated with the plasticity of physiological adaptations to environmental variation. For semi-arid shrublands with a legacy of saline soils, characterization of soil water-tolerant shrub species is necessary for habitat restoration given future projection of increased drought magnitude and persistence in these ecosystems. Five dominant native shrub species commonly found in the Lower Rio Grande Valley, TX, USA, were studied, namely *Acacia farnesiana*, *Celtis ehrenbergiana*, *Forestiera angustifolia*, *Parkinsonia aculeata* and *Prosopis glandulosa*. To simulate drought conditions, we suspended watering of healthy, greenhouse-grown plants for 4 weeks. Effects of soil salinity were also studied by dosing plants with 10% NaCl solution with suspended watering. For soil water deficit treatment, the soil water potential of *P. glandulosa* was the highest (−1.20 MPa), followed by *A. farnesiana* (−4.69 MPa), *P. aculeata* (−5.39 MPa), *F. angustifolia* (−6.20 MPa) and *C. ehrenbergiana* (−10.02 MPa). For the soil salinity treatment, *P. glandulosa* also had the highest soil water potential value (−1.60 MPa), followed by *C. ehrenbergiana* (−1.70 MPa), *A. farnesiana* (−1.84 MPa), *P. aculeata* (−2.04 MPa) and *F. angustifolia* (−6.99 MPa). Within the species, only *C. ehrenbergiana* and *F. angustifolia* for soil water deficit treatment and *A. farnesiana* for the salinity treatment had significantly lower soil water potential after 4 weeks of treatment (*P* < 0.05). We found that soil water potential, stomatal conductance and net photosynthesis of the species significantly reduced over time for both treatments (*P* < 0.05). We conclude that while all species exhibited capacities to withstand current water availability, some species demonstrated limited tolerance for extreme water stress that may be important for management of future shrub diversity in Lower Rio Grande Valley.

## Abstract (precis)

Restoration of degraded shrublands using native shrubs is a challenging task for conservation and management with continued climate change. Investigation of native shrub species from the Lower Rio Grande Valley, USA, showed physiological tolerance to potential future extreme soil water stress though with a gradient of water use strategies that are species specific.

## Introduction

Continued climate change, including increased temperature and decreased precipitation, may have rapid, widespread and long-lasting impacts on species composition and distribution in shrublands (Condit *et al.*, 1995; McDowell *et al.*, 2008; White *et al.*, 2008). Further climate-imposed reduction in soil water availability is likely to intensify with changes in growth, survival and distribution of shrub species in these ecosystems (Grime, 1977; Archer, 1989; González-Rodríguez *et al.*, 2004; Otieno *et al.*, 2005; Gebrekirstos *et al.*, 2006; Morgan *et al.*, 2011). Water stress affects virtually all the physiological functions of plants related to photosynthesis and carbohydrate metabolism that affect their adaptation, growth and distribution (Grime 1977; Escos *et al.*, 2000; Ditmarova *et al.*, 2009). Co-existence of diverse shrub species in shrublands reflects adaptation plasticity with resource partitioning strategies among these species to cope with limited soil water availability (González-Rodríguez *et al.*, 2004); therefore, shrub species in these ecosystems have evolved key morphological and physiological attributes required for adaptation to soil water deficit.

The suitability of shrub species for restoration and conservation of shrublands is related to water stress physiology of target plant species (Gebrekirstos *et al.*, 2006). Plant water relation measures, such as water potential, gas exchange and intrinsic water use efficiency, are common physiological indices to determine the water stress and its effects on plants (Vertovec *et al.*, 2001). Water constraints in shrublands are primarily affected by precipitation inputs and the evaporative environment. Excessive salt in soils also affects the water available to plants by decreasing the osmotic potential of the soil water. Irrigation from surface water increases the soil salt concentration because exclusion of salts by roots during transpiration coupled with low soil water recharge increases the salt concentration in the plant's root zone (McCoy, 1990; Archibald *et al.*, 2006; Wiedenfeld, 2008). A high salt concentration in the soil negatively affects plant water uptake and maintenance of turgor (Maas and Hoffman, 1977).

In this study, we conducted a greenhouse study of five native, dominant shrub species from the Lower Rio Grande Valley (LRGV) of Texas to assess the effects of drought and soil salinity on soil water potential and gas-exchange properties. Within the LRGV, >95% of its original shrub cover was historically removed due to agricultural expansion, fuel wood harvest and other anthropogenic activities during the past century (Ewing and Best, 2004). Restoration at LRGV by land managers during the last two decades has resulted in limited replantation of native shrubs. The restoration programme is also aiming for the conservation of habitat of many endangered species, such as ocelots (*Leopardus pardalis*) and jaguarundis (*Puma yagouaroundi*), which use the dense shrubs as hiding cover. In addition to habitat, these shrublands are important carbon sinks (Navar-Chaidez, 2008). However, successful restoration of this shrubland requires an enhanced understanding of the limitations, tolerances and physiological responses to environmental factors of shrub species to avoid increased soil water deficit. The specific objectives of this study were as follows: (i) to compare the shrub species for their adaptation and tolerance to prolonged soil water stress imposed by soil water deficit and increased soil salinity; and (ii) to assess the conservation of water related to assimilation of carbon as a potential measure of water stress adaptation.

## Materials and methods

### Description of native shrub habitat

The LRGV is located in South Texas, USA, a part of the northern edge of the Tamaulipan Biotic province, which is an assemblage of unique shrubland ecosystems that extends from the Gulf of Mexico plains in south Texas to northern Mexico (Jahrsdoerfer and Leslie, 1988; Reid *et al.*, 1990; González-Rodríguez *et al.*, 2004). Soils of LRGV range from loamy fine sands to clays with deep, moderately fine textures formed in alluvial sediments (Jahrsdoerfer and Leslie, 1988; Wiedenfeld, 2008). Sea salt spray and irrigation water from the Rio Grande are the main sources of soil salinity in this site (Hendrickx *et al.*, 1999; Foltescu *et al.*, 2005; Wiedenfeld, 2008). The soil of LRGV contains between 800 and 900 mg l^−1^ total salt (Wiedenfeld, 2008). The climate is semi-arid and sub-tropical, with long, hot summers and short, mild winters (Eddy and Judd, 2003) with a mean of 330 frost-free days per year. The LRGV receives an annual average rainfall of 68.2 cm, with peak precipitation during September and October (Eddy and Judd, 2003). The yearly potential evapotranspiration is about 220 cm, with average mid-summer vapour pressure deficit values of 3.05 kPa (González-Rodríguez *et al.*, 2004).

### Greenhouse experiment

In order to assess species responses to soil water deficit and soil salinity, we chose five dominant native shrub species from the LRGV for greenhouse study. We cultivated plants of *Acacia farnesiana* (L.) Willd. [Fabaceae], *Celtis ehrenbergiana* (Klotzsch) Liebm. [Ulmaceae], *Forestiera angustifolia* Torr. [Oleaceae], *Parkinsonia aculeata* L. [Fabaceaa] and *Prosopis glandulosa* Torr. [Fabaceae] grown from seeds acquired from LRGV in a greenhouse.

Twelve plants (0.5–1.2 m in height) of each species were grown in 5 l pots filled with commercial potting soil and watered by drip-irrigation every day. Each plant was fertilized with commercial fertilizer (∼3.5 g, Osmocote NPK – 18:6:10; Scotts-Siera Horticultural Product Company, Marysville, OH, USA) added to each pot every 3 months. Out of the 12 plants per species, four plants of each species were randomly selected to be used as controls, with continued daily irrigation. Four of the remaining plants were exposed to soil water deficit treatment by suspension of watering for 4 weeks, referred to as the soil water deficit (SWD) treatment. The remaining four plants of each species were subjected to suspended water for 4 weeks coupled with soil salt treatments, referred to as the salinity treatment. The salt treatment consisted of initial dosing of the potting soil with 680 ml of 0.86 m NaCl to acclimate the plant to the added salt. Next, plants were then irrigated with the addition of 680 ml of 1.72 m NaCl at the beginning of the second week of the experiment. The higher concentration of NaCl solution was equivalent to twice the amount of salt currently present in LRGV soil. In order to avoid biases associated with potential irradiance levels in the greenhouse, the plants were repositioned within the greenhouse each week. The temperature of the greenhouse was maintained at ∼22°C, with relative humidity of 70–90%.

### Soil water potential measurements

The soil water potential (SWP) of each plant pot exposed to the SWD and salinity treatments was measured in continuous mode using a WP4-T Dew Point Potentiameter (Decagon Devices Inc. 2007, Pullman, WA, USA) every week for 4 weeks from the beginning of treatments (from 28 December 2011 to 25 January 2012). Each sample was prepared with ∼8.00 g soil taken at predawn, 8–10 cm below the top of each individual plant pot.

### Gas-exchange measurements

Measurements of midday net photosynthetic rate (*P*_n_) and stomatal conductance (*g*_s_) of water vapour were taken for each plant using a CI-340 Hand-Held Photosynthetic System (CID Inc., 2008, Camas, WA, USA) at midday after SWP measurement. The intensity of photosynthetic active radiation was maintained above 800 μmol m^−2^ s^−1^ during the gas-exchange measurements, because plants grown in open environments require this amount of photosynthetic active radiation for active photosynthesis. For each leaf sample measured, leaf area was measured using Adobe Acrobat Professional 8 (Adobe Systems Inc., 2011) after digitally scanning each leaf (Visoneer OneTouch 9420 Scanner). The leaf area was used to calculate *g*_s_ and *P*_n_ per unit area.

### Statistical analysis

We used repeated-measures ANOVA to determine the effects of species, time, treatments and their interactions on SWP, *g*_s_ and *P*_n_, followed by Tukey's *post hoc* multiple comparisons to analyse the differences between the species and between the time points within the treatments. Pair-wise comparison was used to analyse the differences within the species and between the treatments (control *vs*. SWD or salinity) in terms of SWP, *g*_s_ and *P*_n_. The significance level was set at *P* = 0.05.

## Results

### Soil water potential

**Figure 1.**
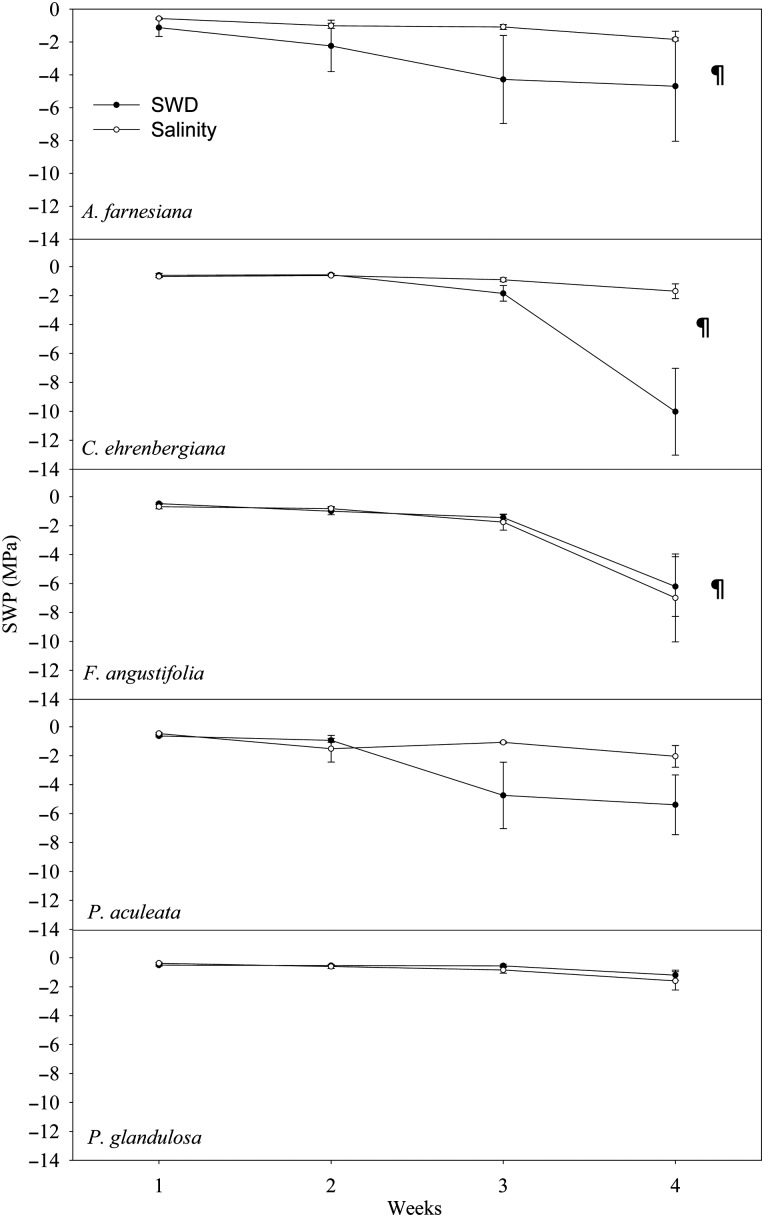
Average soil water potential (SWP) of five native shrub species over 4 weeks for soil water deficit (SWD) and salinity treatments. ¶ Soil of *C. ehrenbergiana* and *F*. *angustifolia* had a significantly lower water potential value at week 4 for both treatments and *A. farnesiana* had a significantly lower water potential at week 4 of salinity treatment.

For the SWD treatment, the water potential of *C. ehrenbergiana* soil in weeks 1 (*P* = 0.004), 2 (*P* = 0.004) and 3 (*P* = 0.012) was significantly higher compared with that in week 4 (Fig. 1). The water potential of *F. angustifolia* soil in weeks 1 (*P* = 0.010), 2 (*P* = 0.019) and 3 (*P* = 0.032) was significantly higher compared with that in week 4 (Fig. 1). For the salinity treatment, the water potential of *A. farnesiana* soil in weeks 1 (*P* = 0.001), 2 (*P* = 0.002) and 3 (*P* = 0.001) was significantly higher compared with that in week 4 (Fig. 1).

The SWP was significantly different among the species for both treatments (Table 1). The SWP of species was significantly reduced by treatment and time for both experiments (Table 1). The interaction between species and treatment, species and time, and time and treatment significantly reduced the SWP of species in salinity treatment (Table 1). The *post hoc* analysis showed that SWP was significantly higher in weeks 1–3 compared with that in week 4 of SWD treatment (*P* = 0.001). During this period, there was a higher SWP in week 1 than in weeks 2 (*P* = 0.008) and 3 (*P* = 0.012). During the 4 weeks of SWD treatment, SWP was significantly higher in week 2 compared with week 3 (*P* = 0.023). For the salinity treatment, weeks 1 (*P* = 0.001), 2 (*P* = 0.001) and 3 (*P* = 0.001) had significantly higher SWP values compared with week 4. During this period, week 1 had significantly higher SWP values compared with week 3 (*P* = 0.001).

**Table 1.** Effects of species, treatments, time and their interactions on soil water potential, photosynthetic rate and stomatal conductance of five native species for control, soil water deficit and salinity experiments

Source	Significance level								
	SWP			*g*_s_			*P*_n_		
	Control	SWD	Salinity	Control	SWD	Salinity	Control	SWD	Salinity
Species	–	0.001	0.001	n.s.	n.s.	n.s.	n.s.	0.024	n.s.
Treatment	–	0.001	0.002	n.s.	0.001	0.001	n.s.	0.001	0.001
Time	–	0.003	0.001	n.s.	0.008	0.004	n.s.	0.001	0.002
Species × treatment	–	n.s.	0.001	n.s.	n.s.	n.s.	n.s.	n.s.	n.s.
Species × time	–	n.s.	0.001	n.s.	n.s.	0.002	n.s.	n.s.	n.s.
Treatment × time	–	n.s.	0.001	n.s.	0.001	n.s.	n.s.	n.s.	n.s.

*P* values were determined by repeated-measures ANOVA at 95% confidence intervals; n.s. represents non-significant results at *P* = 0.05 significance level.

Abbreviations: *g*_s_, stomatal conductance; *P*_n_, photosynthetic rate; SWD, soil water deficit; and SWP, soil water potential.

### Gas exchange

**Figure 2.**
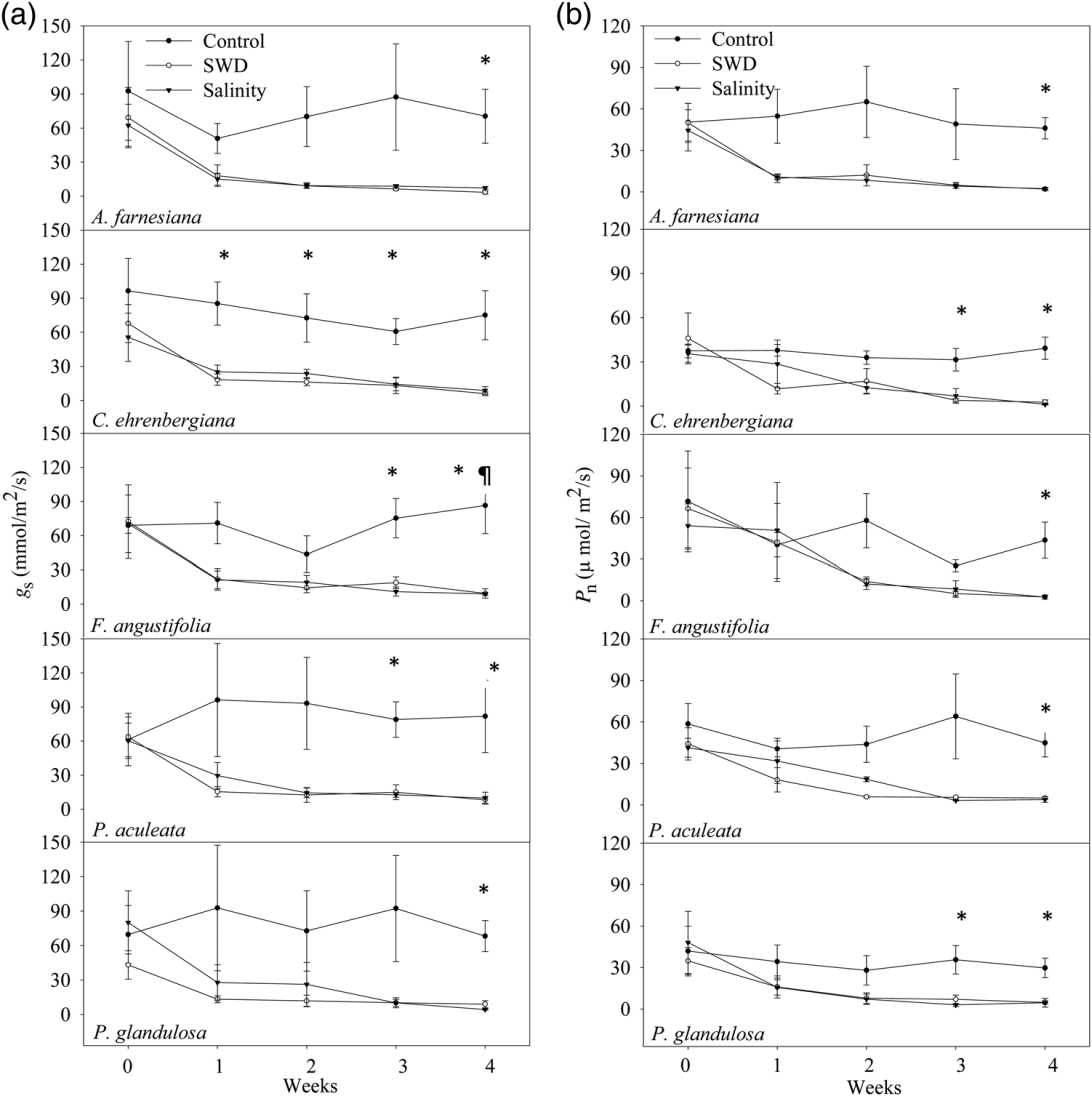
Average values of stomatal conductance (*g*_s_; **a**) and photosynthetic rate (*P*_n_; **b**) for five native shrub species with 95% confidence intervals showing the result of control, SWD and salinity treatments, respectively. * Significantly lower *g*_s_ and *P*_n_ of each species compared with its control plants for SWD and salinity treatments. ¶ Significantly lower *g*_s_ value of *F. angustifolia* for salinity treatment only at week 4.

The average *g*_s_ value of control plants of all species remained constant throughout the experiment, with no significant difference between species or over time. For the SWD treatment, *A. farnesiana* had significantly lower *g*_s_ than control plants at week 4 (*P* = 0.023; Fig. 2a). *Celtis ehrenbergiana* had significantly lower *g*_s_ values compared with control plants at weeks 1 (*P* = 0.008), 2 (*P* = 0.028), 3 (*P* = 0.001) and 4 (*P* = 0.01; Fig. 2a). Compared with control plants, *F. angustifolia* had significantly lower *g*_s_ at weeks 3 (*P* = 0.012) and 4 (*P* = 0.027; Fig. 2a). At weeks 3 (*P* = 0.003) and 4 (*P* = 0.011), *P. aculeata* had significantly lower *g*_s_ values than that of control plants (Fig. 2a). When compared with control plants, *P. glandulosa* had significantly lower *g*_s_ at week 4 (*P* = 0.025; Fig. 2a).

For the salinity treatment, *A. farnesiana* had significantly lower *g*_s_ values compared with that of control plants at week 4 (*P* = 0.012; Fig. 2a). When compared with control plants, *C. ehrenbergiana* had significantly lower *g*_s_ values at weeks 1 (*P* = 0.015), 2 (*P* = 0.049), 3 (*P* = 0.001) and 4 (*P* = 0.012; Fig. 2a). At week 4, *F. angustifolia* had significantly lower *g*_s_ values than that of control plants (*P* = 0.022; Fig. 2a). At weeks 3 (*P* = 0.012) and 4 (*P* = 0.003), *P. aculeata* had significantly lower *g*_s_ values compared with control plants (Fig. 2a). Compared with control plants, *P. glandulosa* had significantly lower *g*_s_ at week 4 (*P* = 0.005, Fig. 2a).

The *g*_s_ values of the species were significantly reduced when analysed by treatment and time for SWD and salinity treatments (Table 1). The interaction of time and treatment significantly reduced the *g*_s_ of the species for SWD treatment (Table 1). The interaction of species and time had significant effects on *g*_s_ of the species for salinity treatment (Table 1). The *post hoc* analysis showed that *g*_s_ of the species was significantly reduced over time; week 0 had significantly higher values compared with those of weeks 1 (*P* = 0.001), 2 (*P* = 0.001), 3 (*P* = 0.001) and 4 of SWD (*P* = 0.001). There were significantly higher *g*_s_ values in week 1 (*P* = 0.007) than in week 4 (*P* = 0.024) during this period of SWD treatment. For the salinity treatment, there was a significantly higher *g*_s_ value in week 0 (*P* = 0.001) than in weeks 1 (*P* = 0.001), 2 (*P* = 0.002), 3 (*P* = 0.001) and 4 (*P* = 0.001). During this period, there was a significantly higher *g*_s_ value in week 1 than in weeks 3 (*P* = 0.023) and 4 (*P* = 0.005) of the salinity treatment.

The average *P*_n_ value of all control plants remained unchanged throughout the experiment, with no significant difference between species or over time. For the SWD treatment, *A. farnesiana* (*P* = 0.001), *F. angustifolia* (*P* = 0.029) and *P. aculeata* (*P* = 0.003) had significantly lower *P*_n_ values at week 4 compared with that of control plants (Fig. 2b). *Celtis ehrenbergiana* had significantly lower *P*_n_ values at weeks 3 (*P* = 0.013) and 4 (*P* = 0.001) compared with control plants (Fig. 2b). At week 3 (*P* = 0.024) and 4 (*P* = 0.009), *P. glandulosa* had significantly lower *P*_n_ values compared with that of the control plants (Fig. 2b).

For the salinity treatment, we found that *A. farnesiana* (*P* = 0.001), *F. angustifolia* (*P* = 0.018) and *P. aculeata* (*P* = 0.003) had significantly lower *P*_n_ values at week 4 compared with the control plants (Fig. 2b). At weeks 3 and 4, *C. ehrenbergiana* had significantly lower *P*_n_ values (*P* = 0.03 and *P* = 0.013, respectively) compared with the control plants (Fig. 2b). At weeks 3 (*P* = 0.012) and 4 (*P* = 0.008), *P. glandulosa* had significantly lower *P*_n_ values compared with the control plants (Fig. 2b).

The *P*_n_ was significantly different only among the plant species in the SWD treatment (Table 1). The *P*_n_ of species was significantly reduced by treatment and time for SWD and salinity treatments (Table 1). The *post hoc* analysis showed that there was a significantly higher *P*_n_ of the species in week 0 than in weeks 1 (*P* = 0.003), 2 (*P* = 0.002), 3 (*P* = 0.001) and 4 (*P* = 0.001) for the SWD treatment. For the salinity treatment, there was a significantly higher *P*_n_ in week 0 compared with weeks 2 (*P* = 0.001), 3 (*P* = 0.001) and 4 (*P* = 0.001). During this period, there was a significantly higher *P*_n_ value in week 1 than in weeks 3 (*P* = 0.046) and 4 (*P* = 0.009). During the 4 weeks of salinity treatment, there was a significantly higher *P*_n_ value in week 2 than in week 4 (*P* = 0.001).

## Discussion

The significant reduction of SWP values during the experiment due to the effects of drought and soil salinity indicates that soil water availability to the plants was reduced over time. The experiment showed that shrub species can survive with a soil water potential as low as −10.02 MPa (e.g. *C. ehrenbergiana*). Plant species that continue to function with a higher SWP may have specific adaptations to rehydrate plant cells more efficiently from available soil water or minimize the transpiration rate to conserve available soil moisture (Ritchie and Hinckley, 1975; Wan and Sosebee, 1991). Several studies have shown that SWP equilibrates with leaf water potential due to low atmospheric demand for water during the pre-dawn period (Morgan, 1984; Saliendra *et al.*, 1995; Sellin, 1996; González-Rodríguez *et al.*, 2000; Gebrekirstos *et al.*, 2006). However, areas with high minimal temperatures may have transpiration at night where pre-dawn leaf water potential does not equilibrate with SWP (Donovan *et al.*, 1999, 2001, 2003; Bucci *et al.*, 2004; Howard *et al.*, 2009). Other factors responsible for this disequilibrium may include low osmotic potential of plant species (Berger *et al.*, 1996), hydraulic resistance to the soil–plant pathway (Myers and Neales, 1984), smaller plant size (Brown and Archer, 1990), night-time growth-induced reduction in cell water potential (Boyer, 1995) and soil moisture heterogeneity (Ourcival *et al.*, 1994).

Based on the results of observed SWP, we categorized *A. farnesiana*, *C. ehrenbergiana* and *F. angustifolia* as water stress-sensitive species, because the soil of these species significantly reduced the water potential during the experiments. On the contrary, *P. aculeata* and *P. glandulosa* were categorized as water stress-tolerant species, because we did not observe significant loss of soil water potential for these species. For *C. ehrenbergiana*, the large decrease in SWP may be associated with high transpiration of this species, which also had higher initial stomatal conductance. Hence, plants that can reduce transpiration during drought may have higher survivorship in comparison to the species with higher transpiration rates. Soil water stress-tolerant species can withstand extreme dehydration of the protoplasm (González-Rodríguez *et al.*, 2011). These species continue to maintain gas exchange under strongly negative plant water potential by maintaining osmotic potentials or by accumulating solutes or by both mechanisms (Morgan, 1984; Gebrehiwot *et al.*, 2005).

Higher SWP with saline soils suggests that salinity may reduce water loss from species during a prolong water stress period. Higher SWP during soil water stress suggests that solute concentration in the root zone may reduce water loss from plants during relatively longer periods of water stress (Kozlowski, 1997). Salt accumulation in the root zone of these species may result in an increased solute concentration in the xylem content due to mobilization of cell sap from cells en route to reduce water loss, resulting in inefficient water extraction from the soil (Gollan *et al.*, 1985; González-Rodríguez *et al.*, 2011). The species grown in soil with higher water potential can maintain higher plant water potential compared with other coexisting species in an identical environment that might involve osmotic adjustments during the soil water stress (Morgan, 1984; Tezara *et al.*, 1999; Chaves *et al.*, 2003). Such species tolerate soil salinity by absorbing ions from the soil that are sequestered in cell vacuoles or synthesized into compatible solute in the cytoplasm (Kozlowski, 1997). Significantly lower SWP in *F. angustifolia* and higher SWP in *P. glandulosa* compared with the other species after 4 weeks of the salinity treatments may be connected to higher and lower transpiration rates, respectively, for these species.

In the field, water stress-tolerant species may be phreatophytic by necessity. For example, *P. glandulosa* may extract water from soil through a deep rooting system that differentiates it from species with a shallow rooting system (e.g. *A. farnesiana*, *C. ehrenbergiana* and *F. angustifolia*). Plant strategies to cope with water stress may be related to a deep *vs*. shallow rooting system in competitive field environments (González-Rodríguez *et al.*, 2011). The advantage of allocating biomass in deep root systems by the phreatophytic species may be to avoid competition for available water in the upper soil surface with shallow-rooted species. However, the greenhouse study showed that water stress-tolerant responses are related to the water conservation strategy rather than the phreatophytic characteristics. Such species have the capacity to reduce the transpiration rate with decreasing soil water availability (Wan and Sosebee, 1991). These characteristics of *P. glandulosa* partly explain its increased dominance in drought-prone shrublands of Texas over the past century (Brown and Archer, 1999).

The findings of this study showed that a significant reduction in *g*_s_ occurred earlier than in SWP of some species. This suggests that a small reduction in the available soil water affects gas exchange of these plants. Mechanisms for this may be excessive root production of abscisic acid during drought, which is delivered to leaves by transpirative flow and triggers stomatal closure to avoid tissue dehydration (Gowing *et al.*, 1993; Trejo *et al.*, 1993; Montagu and Woo, 1999). The complex interactions of other environmental factors, such as leaf water potential, xylem hydraulic conductivity, plant nutritional status, xylem sap pH and leaf-to-air vapour pressure deficit, were also reported to influence stomatal control during soil water stress (Dai *et al.*, 1992; Tardieu and Davies, 1992; Salleo *et al.*, 2000; Medrano *et al.*, 2002). The salt concentration in the rooting zone reduces root hydraulic conductance; hence, it reduces the amount of water flow from the roots to the upper portion of the canopy, causing a reduction in *g*_s_ (González-Rodríguez *et al.*, 2004). The SWP can be even lower in the field compared with a controlled environment that may increase variation in the stomatal response to imposed drought effects (Davies, 1977; Dawyer and Stewart, 1985).

We also found that *g*_s_ was affected by each of the treatments ∼1 week earlier than that of net photosynthesis in three of the species, suggesting that soil water stress affects water vapour conductance independent of photosynthesis. Plants may show both stomatal and non-stomatal limitation of photosynthesis during water stress (Noormets *et al.*, 2001). *Acacia farnesiana* demonstrated stomatal control of photosynthesis, where lower *g*_s_ and photosynthesis occurred in the same week for this species. For the remaining species, our data suggest non-stomatal inhibition of photosynthesis due to increased CO_2_ reduction in the chloroplast, as well as reduced efficiency of ribulose biphosphate regeneration due to inactivated electron transport via shrinkage of intercellular spaces (Iyengar and Reddy, 1996). The *g*_s_ of most species decreases with high salinity, as we observed (Parida *et al.*, 2004), which is likely to induce closure of stomata with restricted availability of internal CO_2_ for carboxylation (Brugnoli and Björkman, 1992). Also, the leaf area changes associated with loss of turgor may affect cell wall properties, with a decrease in net photosynthesis being associated with deformation of the cellular structure (Franco *et al.*, 1997; González-Rodríguez *et al.*, 2004). However, more investigation is required to determine how stomatal or non-stomatal reductions in photosynthesis occur for the shrub species tested in this study.

We found significantly reduced values of *P*_n_ of all species that occurred before decreases in SWP values in both treatments. In a resource-limited ecosystem, low accumulation of water and minerals available for investment in the photosynthetic apparatus may significantly reduce the photosynthesis of species that maintain a higher plant water potential (Soyza *et al.*, 2004). Water stress imposed due to prolonged soil water deficit and soil salinity may affect the growth and survival of plants due to a reduction in gas exchange, although this may vary greatly among species (Bolarín *et al.*, 1991). A potential reason for the lower photosynthesis found due to the treatments might be reduced ATP synthesis, with overall lower rates of cellular metabolism (Tezara *et al.*, 1999). Reduction in photosynthesis due to soil salinity is accompanied by dysfunction in protein and nucleic acid metabolism and enzymatic activities (Kozlowski and Pallardy, 2002). However, we found no difference in net photosynthesis among the species for both treatments, indicating that the gas-exchange capacity of these species is broadly affected by soil water deficit.

Only seedlings of the plant species were tested in this experiment. Some research indicates that growth form and plant size may influence the physiological responses of species to environmental factors. For example, *P*_n_ and *g*_s_ of seedlings showed lower values compared with larger plants of the same species at more arid locations (Brown and Archer, 1990; Knapp and Fahnestock, 1990; Donavan and Ehleringer, 1991, 1992). However, seedlings of plant species grown in wet soil may have higher *g*_s_ when compared with larger plants of same species (Donavan and Ehleringer, 1991). The variation may be attributed to the shallow rooting system of younger plants in the field and the smaller taproot for water storage (Brown and Archer, 1990; Knapp and Fahnestock, 1990). Hence, larger plants may show potential to avoid environmental stress that may have stronger and more negative effects on smaller plants of the same species.

In semi-arid shrublands, such as the LRGV, precipitation has been gradually declining over the past century, with increased magnitude and duration of drought periods (Porporato *et al.*, 2001). Coupled with inherently saline soils from both the legacy of agricultural irrigation and airborne inputs from the ocean, the inter-specific variations in soil water potential and gas-exchange values recorded during the experiment suggest that the species showed differences in their capacity to withstand a wider range of soil water status. All species studied are potential candidates for continued restoration and conservation of these degraded shrubland ecosystems. However, maintaining future diversity for Tamaulipan shrublands may be difficult given the range of water stress responses shown for this common group of shrubs species.

### Conclusion

We found physiological evidence that the soil water potential of the five co-occurring shrub species decreased in response to progressive water stress, affecting their gas-exchange capacity. Water stress due to drought and salinity affects inter-specific differences in water relation and gas-exchange characteristics of these species. Our findings suggest that the coexisting plants have developed tolerance strategies to soil water stress, but the extent can be species specific. Our results also indicated that soil water potential drives gas-exchange limitation in some species and that photosynthesis in some species may be controlled by non-stomatal mechanisms of tissue water and solute concentration. From our experiments, while all five species exhibited capacities to withstand current water availability, some species have limited tolerances for extreme water stress.
